# Effectiveness of virtual reality exposure therapy in improving body dissatisfaction and shape-related concerns in adolescent girls with anorexia nervosa: a controlled, randomized, superiority trial

**DOI:** 10.1186/s13063-026-09929-4

**Published:** 2026-08-06

**Authors:** Yohann Kierbel, Antoine Manès, Coralie Barbe, Anne-Catherine Rolland, Antoine Stocker, Marilou Lamourette, Julie Rolling, Julien Eutrope

**Affiliations:** 1 Service de Pédopsychiatrie, CHU Reims, Université Reims Champagne-Ardenne, C2S, Reims, France; 2 Service de Pédopsychiatrie, CHU Reims, Reims, France; 3 Comité Universitaire de Ressource Pour La Recherche en Santé, Université Reims Champagne-Ardenne, Reims, France; 4 Service de Pédopsychiatrie, Université Toulouse 3, CERPOP, Inserm, UPS, CHU Toulouse, Toulouse, France; 5 UMR 1319, INSPIIRE, Inserm, Pôle Universitaire de Psychiatrie de L’Enfant Et de L’Adolescent, Centre Psychothérapique de Nancy, Université de Lorraine, Laxou, Nancy, France; 6 Centre Régional du Psychotraumatisme Grand Est, CNRS UPR 3212, Institut Des Neurosciences Cellulaires Et Intégratives, Service de Pédopsychiatrie, Université Strasbourg, CHU Strasbourg, Strasbourg, France

**Keywords:** Adolescents, Anorexia nervosa, Body dissatisfaction, Shape-related concerns, Virtual reality

## Abstract

**Background:**

Anorexia nervosa (AN) involves qualitative and quantitative dietary restrictions, leading to significant weight loss and changes in body shape. Patients with AN are often more concerned about their body image than the general population is. Body dissatisfaction and shape-related concerns (BD-SRC), which are risk factors for relapse, are some of the most difficult AN symptoms to treat. This study aims to assess the effectiveness of Virtual Reality Exposure Therapy (VRET) in improving BD-SRC in adolescent girls with AN.

**Method:**

This prospective, multicenter study will be a controlled, randomized, superiority trial. This study will be an open-ended trial. In total, 40 patients with AN will be randomly assigned to the “VRET” or the “body-first psychomotricity” group. Adolescent girls will be randomly divided into two groups: 20 each receiving VRET and 20 receiving treatment with body-first psychomotricity treatment. The primary objective will be rated 12 months after randomization using the Body Shape Questionnaire.

**Discussion:**

The study hypothesizes that VRET is more effective than a body-first psychomotricity approach in improving BD-SRC and that this improvement is stable in the long term, even after treatment has stopped. If this hypothesis is confirmed, VRET could offer an interesting new therapeutic alternative to the traditional European approach of body-first psychomotricity for adolescent girls with AN.

**Trial registration:**

Trial registration: ClinicalTrials, NCT04107870, Registered on September 26, 2019. https://clinicaltrials.gov/show/NCT04107870.

**Supplementary Information:**

The online version contains supplementary material available at https://doi.org/10.1186/s13063-026-09929-4.

## Background

Anorexia nervosa (AN) is a severe psychiatric disorder that generally affects young girls, with a sex ratio of nine girls to one boy [1]. Girls aged 10–14 have seen a significant increase in the incidence of AN among girls studies [2]. The highest rates of the condition were observed among individuals aged 15–19 [3].

Recent systematic reviews have estimated the worldwide prevalence of AN in children and adolescents to be approximately 0.6%, with significantly higher rates in girls than in boys [4]. A 2022 study by Silén and Keski-Rahkonen showed an increase in AN prevalence since the COVID-19 pandemic [5]. Notably, AN is one of the most lethal psychiatric illnesses worldwide. The estimated aggregate mortality rate per decade is 5.6%, with 50% of cases resulting from cardiac arrest and 50% from suicide [3, 6]. All treatments should be provided on an outpatient basis, except in cases of physical or psychiatric health emergency [7]. Therefore, a global assessment of the patient (physical, nutritional, and psychological) must be performed to evaluate the intensity of the symptoms and signs of physical or psychiatric severity that could justify hospitalization. Social and family dynamics are also important in the evaluation of signs of severity.

Bruch [8] first recognized the association between changes in body image and AN in the early 1960s. Patients with AN are obsessed with their weight and thinness and display compulsive behaviors. Moreover, patients with AN may lack awareness of their disorder, with distorted cognition of having too much body fat, despite being extremely thin. They resort to extreme solutions and place their lives in danger of their ideal of thinness. Body-image disturbances include various psychological issues. These range from general dissatisfaction with and negative emotions about one’s body to excessive concern about one’s appearance. These issues exist on a continuum ranging from body dissatisfaction and shape-related concerns (BD-SRC) to the severe distress experienced in body dysmorphic disorders [9]. This criterion has become one of the key criteria for diagnosing AN in the DSM-5, in its first and revised editions [10, 11].

1. Restriction of energy intake relative to requirements leads to a significantly lower body weight in the context of age, sex, developmental trajectory, and physical health [10, 11].
2. Intense fear of gaining weight or becoming fat, or persistent behavior, interferes with weight gain, even at a significantly low weight [10, 11].
3. Disturbances in the way in which one’s body weight or shape is experienced, undue influence of body weight or shape on self-evaluation, or persistent lack of recognition of the seriousness of current low body weight [10, 11].

AN can be categorized as restrictive or binge eating or purging. The restrictive type was characterized by not regularly engaging in binge eating or purging over the last 3 months. The binge eating/purging type is defined as regularly engaging in binge eating or purging over the last three months [11].

Recent studies have shown that adolescent girls with AN exhibit pronounced BD-SRC, which is characterized by negative body image, body-checking behaviors, and distorted body perception. BD-SRC acts as a risk factor and a perpetuating factor of this disorder [12]. Given the role of BD-SRC in the onset and persistence of AN in adolescent girls, it is important to determine the most appropriate treatment approaches for this condition.

Another widely known aspect of AN is weak central coherence and difficulties in cognitive flexibility, such as set-shifting. Some studies have shown that patients with AN tend to have greater set-shifting difficulties and weaker central coherence, thus impacting body image and shape-related concerns, particularly by causing a rigid preoccupation with thinness, excessive attention to detail, and perfectionistic tendencies. Treatment interventions such as cognitive behavioral therapies have been shown to remediate these biases, thus alleviating body image disturbances [13, 14].

Virtual Reality Exposure Therapy (VRET) is a therapeutic procedure involving the substitution of reality (“in vivo” exposure phases) with artificially created and controlled stimuli in a virtual environment. The VRET has also been evaluated in adults with eating disorders. Several studies using VRET have displayed an improvement in BD-SRC and desire to change compared to psycho-nutritional or cognitive behavioral therapy treatment alone [15–17]. In a study by Marco, Perpiñá, and Botella [18] that compared two groups of adult patients with EDs, a significant reduction in BD-SRC was observed in the group that received VRET compared to the group that only received cognitive behavioral therapy. Recent studies comparing changes in the BD-SRC among patients with AN (adolescents and adults) who received treatment as usual or VRET have shown that the “VRET group” experienced a significantly greater reduction in these symptoms [19, 20].

Body-oriented psychotherapy is an umbrella term for approaches that use physical techniques to enhance the therapeutic dialogue on experiences and perceptions. The body serves as the primary means of communication. These methods are particularly effective in treating psychosomatic conditions such as eating disorders (Complementary Therapies in Clinical Practice, 2025). Psychomotricity is a holistic, body-oriented therapeutic approach that integrates motor, cognitive, and emotional processes, as defined by the European Forum of Psychomotricity, and is closely related to the psychomotor therapy used in several European countries [21, 22]. In this approach, the psychomotrician uses mediation involving the body to remedy difficulties or disorders. In psychocorporal care for patients with AN, the aim is to help them develop a body image by paying close attention to their sensations and perceptions, which are closely linked to their emotional experiences. The body psychotherapy approach enables patients who have progressively disengaged from their bodies to reconnect with their experiences, sensations, and perceptions, thus facilitating the reappropriation of the body and abolition of BD-SRC symptoms. The main themes of their misperceptions of the body do not concern weight, as one might expect, but rather the shape and limits/contours of their body, and more specifically, certain parts (stomach, thighs, buttocks, etc.). Psychomotor work with patients with AN was based on the work of Wilhelm Reich [23]. It focuses on the posture, breathing, muscular tension, and body awareness [24]. This type of body-oriented therapy has been shown to be effective as part of a holistic approach to the management of eating disorders in AN, notably reducing the intensity of BD-SRC symptoms [25].

## Objectives

### Primary objective

The primary objective of this study will be to evaluate the effectiveness of VRET in improving BD-SRC in patients with AN compared to the traditional body-first psychomotricity approach.

### Secondary objectives

The secondary objectives of this study will be as follows: (1) to study the long-term outcome (12 months after completing treatment) of patients diagnosed with AN; and (2) to study, among patients with AN, the association between the evolution of BD-SRC and the evolution of emaciation via the median body mass index (BMI) percentage (% m-BMI), symptoms of anxiety as evaluated by the Spielberger State-Trait Anxiety Inventory (STAI), central coherence, as evaluated by the Rey–Osterrieth Complex Figure Test (ROCF) and cognitive flexibility (or shifting) as evaluated by the Trail Making Test (TMT).

## Hypotheses

The hypothesis of this study was that the effectiveness of VRET in improving BD-SRC is superior to a body-first psychomotricity approach, and that this effectiveness tends to have long-term stability, even at 24 months.

## Methods

### Study design

This will be a multicenter, comparative, randomized, open, controlled, superiority trial with prospective inclusion criteria. The list of study sites was obtained from the University Hospital of xxxxx Research and Public Health.

### Randomization

Treatment methods (“VRET” and “body-first psychomotricity approach”) will be randomized by blocks, with a ratio of 1:1. Randomization will be stratified by AN type according to the DSM-5 TR criteria (binge eating/purging or restrictive type), as detailed in the introduction. Randomization was conducted after obtaining informed consent. Randomization will be performed using the CleanWeb software by the physician, including the patient, and the allocation group will be announced immediately after randomization by the same physician.

### Participants

The inclusion criteria will be as follows: (1) female patients diagnosed by a pediatric psychiatrist within the last six months with AN of any type (including purgative and restrictive) according to the DSM-5; (2) 13–17 years old; (3) native French speakers; (4) voluntary agreement to participate in the study; (5) parents or guardians who consented to participate; and (6) registered with a social security regime.

Patients (1) with any psychotic disorder (diagnosed by the Mini International Neuropsychiatric Interview for Children and Adolescents [MINI-KID) corresponding to the schizophrenia spectrum and other psychotic disorder categories in the DSM 5-TR), (2) with an addiction to any psychoactive substance (diagnosed by MINI-KID), (3) with criteria of AN somatic severity, or (4) receiving treatment with benzodiazepines will be excluded. Moreover, participants requiring hospitalization or resuscitation owing to medical complications related to the AN during the study will be retrospectively excluded.

To verify that the patients met the inclusion criteria, a biological check-up will be arranged if one had not been performed within the last 7 days. The criteria for somatic severity were as follows.

- Complete food refusal
  BMI < 14 kg/m^2^ in individuals over 17 years of age, BMI < 13.2 kg/m^2^ in those aged 15–16 years, and BMI < 12.7 kg/m^2^ in those aged 13–14 years
- Confusion
  Bradycardia < 40 bpm; tachycardia
- Blood pressure < 80/50 mmHg
- Hypothermia < 35.5 °C
- Severe metabolic disturbances: hypokalemia < 2.5 mmol/L, hyponatremia < 125 mmol/L or hypernatremia > 150 mmol/L, hypophosphatemia < 0.5 mmol/L
- Ketonuria, hypoglycemia <0.6 g/L
- Elevated creatinine > 100 µmol/L
- Hepatic cytolysis > 4 times the upper limit of normal (ULN)
- Leukoneutropenia < 1000/mm^3^
- Thrombocytopenia < 60,000/mm^3^
- Suicide attempts within the month preceding inclusion

#### Sample size

The number of participants required was calculated using the primary judgment criterion (evolution of the BSQ score at 12 months post-randomization). Thus, the following hypotheses were proposed:

- Average evolution of 31.9 ± 8.12 for the psychomotricity treatment group. This evolution corresponded to that observed in patients with AN after hospital treatment in a study by Lavoisy et al. [26].
- Greater evolution for the VRET group than for the psychomotoricity treatment group is expected.

With an alpha risk of 5%, power of 90%, and a bilateral test, the number of participants required was estimated to be 15 per group (NQuery 4.0 software). Considering a rate of 20% of patients lost to follow-up, we planned to include 20 patients in each group. Strategies to improve adherence to the intervention protocol have been put in place, such as compensating transportation costs for the first visit and adapting session dates to participants’ schedules while respecting the expected intervals between each session.

Strategies for achieving adequate participant enrollment to reach the target sample size were used, such as the participation of four centers used in common research protocols, choosing patients under long-term care for their pathology, and choosing patients with a high level of interest in the proposed care.

### Recruitment

Participation in the study will be proposed to all eligible patients during their first pediatric or psychiatric consultation. Information regarding the research objectives and methods will be provided to the patient as well as their parents or guardians with the help of an information note. If the patient and parents or guardians agree to the patient’s participation in the study after being given some time to consider, the patient will be included, and an inclusion visit specific to the study will be arranged.

### Procedures

Clinical data will be collected during the inclusion visit, such as sociodemographic data, anthropometric data, and ongoing treatment. Questionnaires specific to the study will be administered to the patients: MINI-KID, BSQ, EDI-2, CDRS, STAI, ROCF, and TMT. Randomization will be performed during the inclusion visit after screening to verify eligibility.

**Fig. 1:**
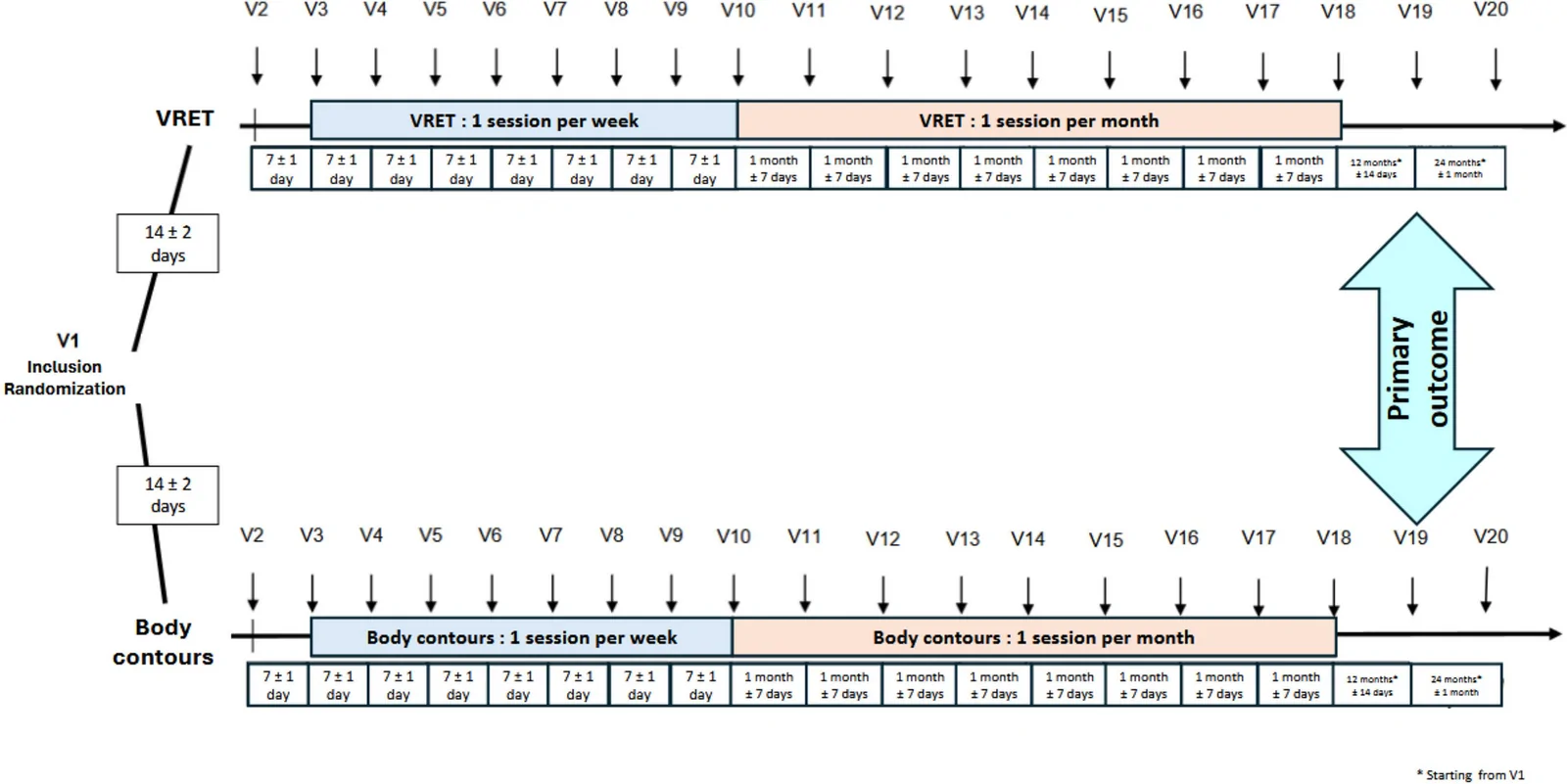
Investigation plan

The investigation plan and data collection are summarized in Table 1 and Fig. 1.

**Table 1:**
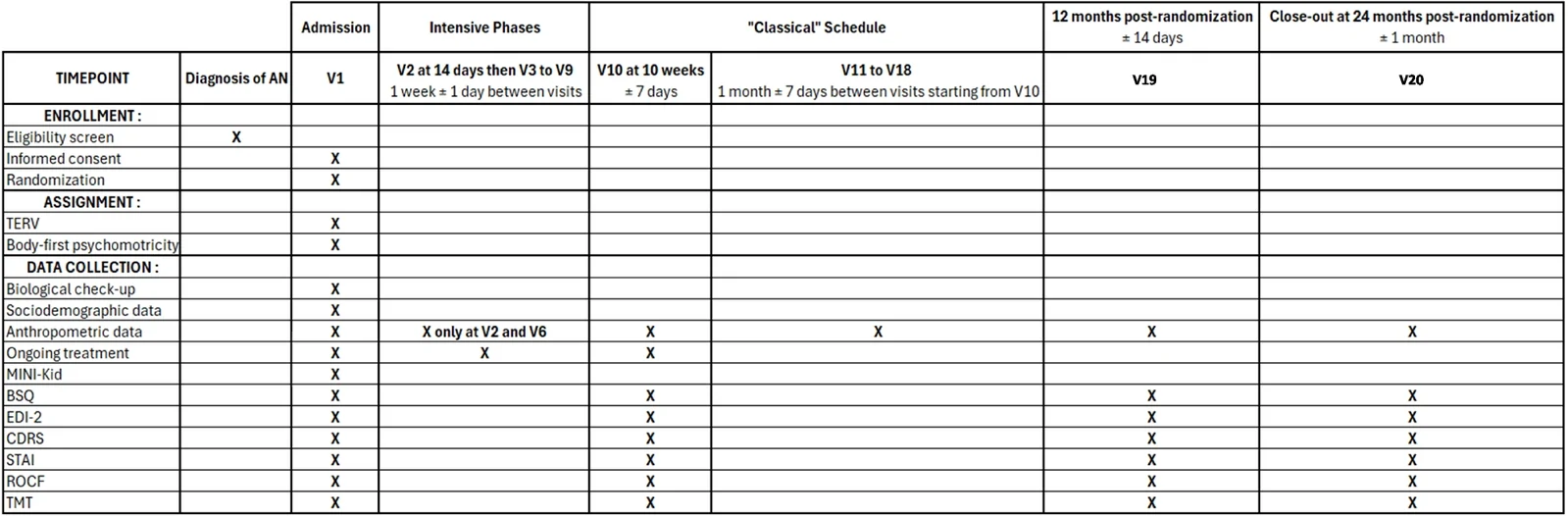
Research schedule

*AN* anorexia nervosa, *V* visits, *TERV* Virtual Reality Exposure Therapy, *BSQ* Body Shape Questionnaire, *EDI* Eating Disorder Inventory, *CDRS* Contour Drawing Rating Scale, *STAI* State-Trait Anxiety Inventory, *ROCF* Rey-Osterrieth Complex Figure Test, *TMT* Trail Making Test

### Intervention

#### Treatment with virtual reality exposure therapy

**Fig. 2:**
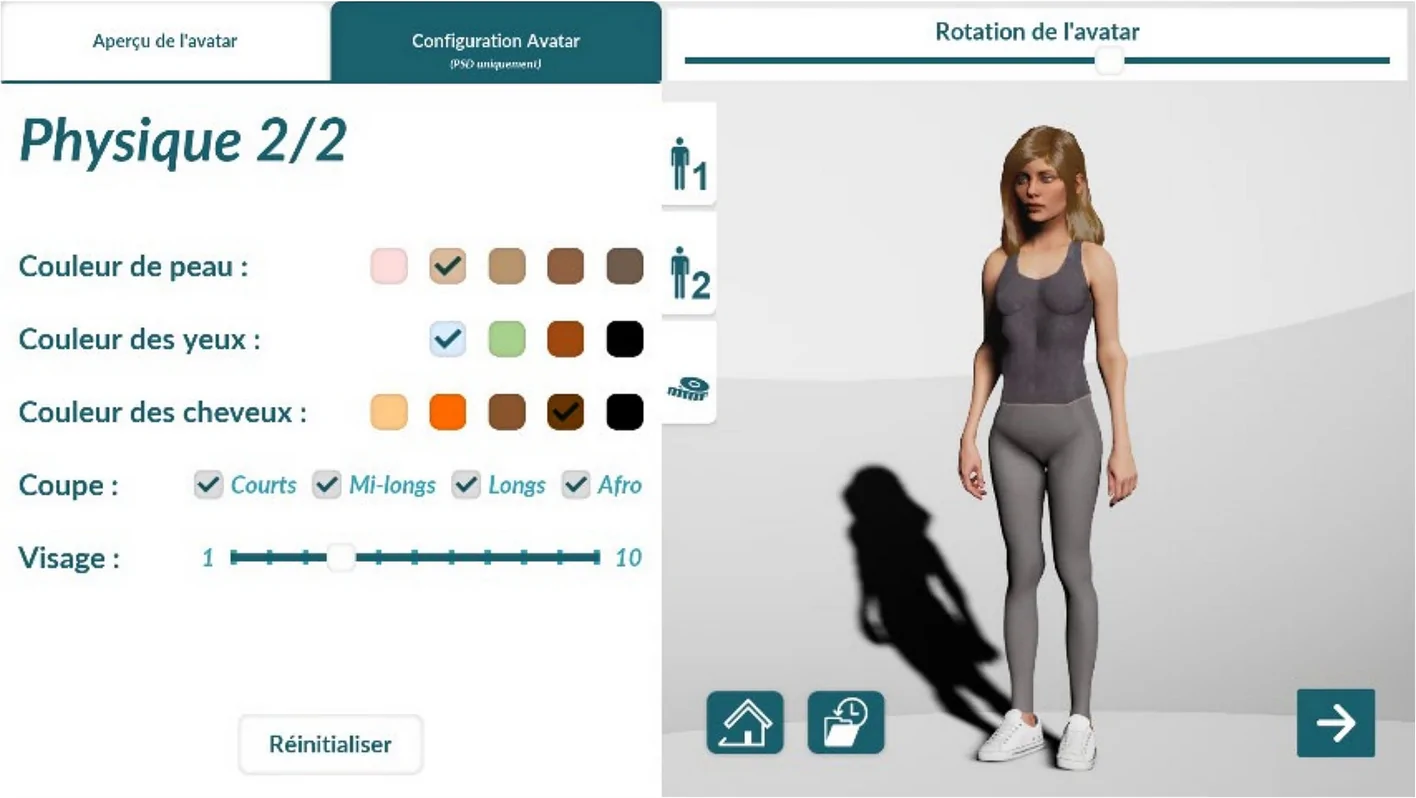
Measurement

**Fig. 3:**
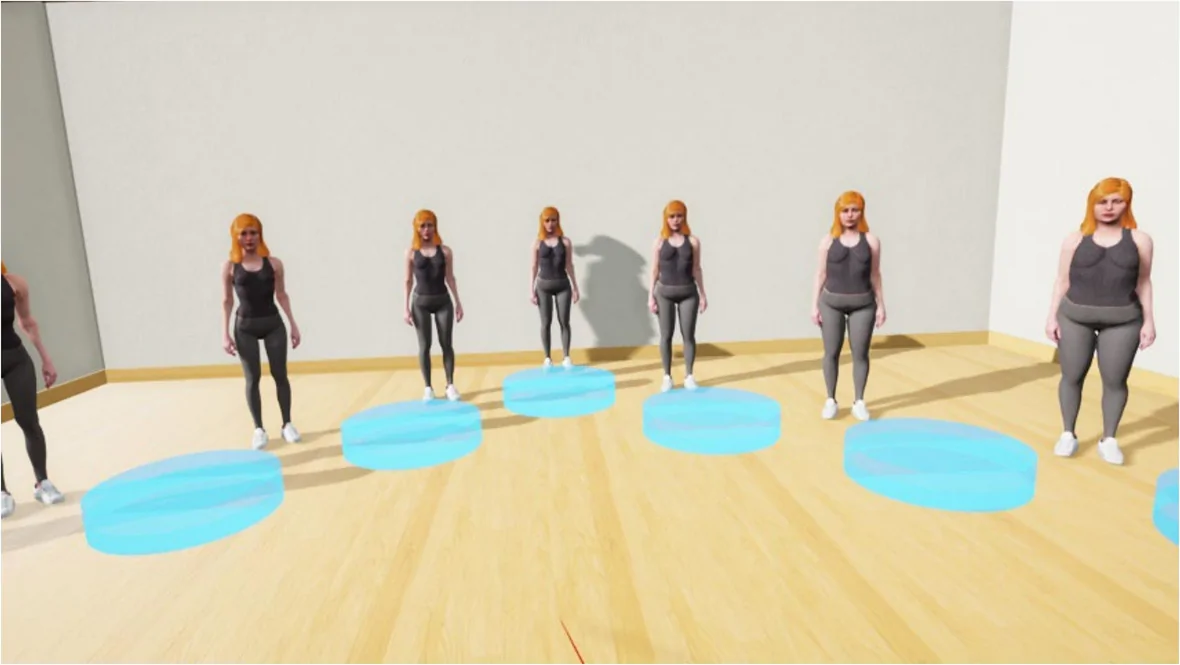
Zone 1

**Fig. 4:**
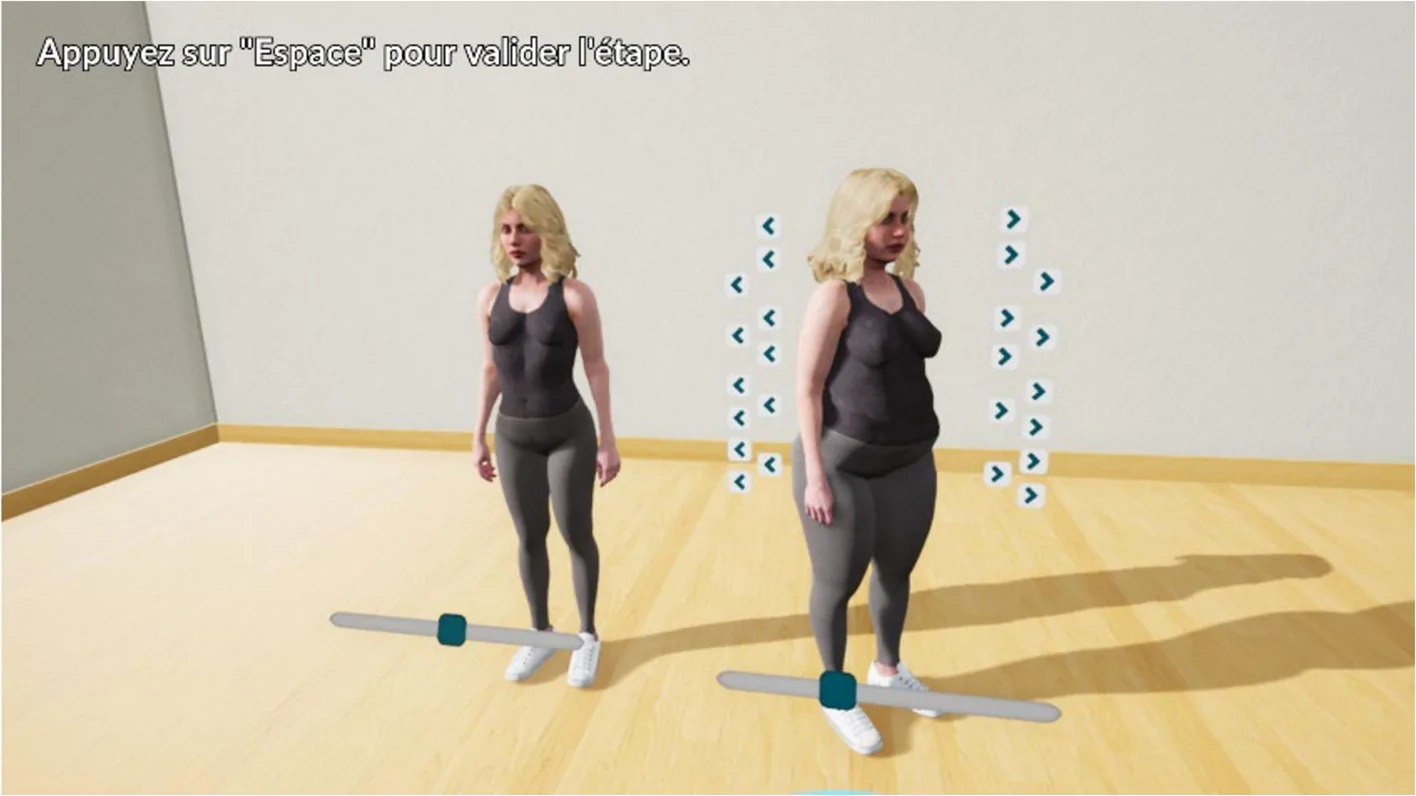
Zone 2

**Fig. 5:**
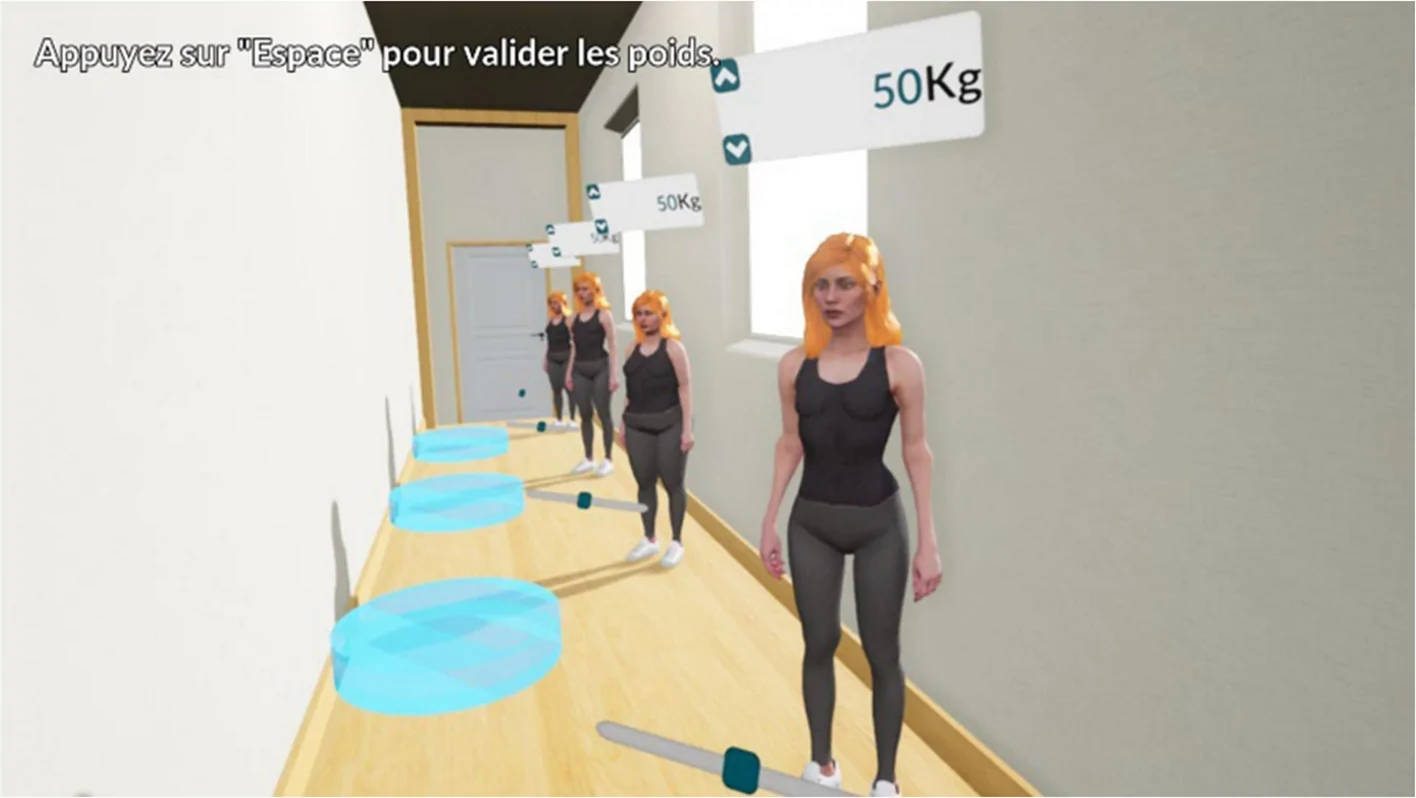
Zone 3

**Fig. 6:**
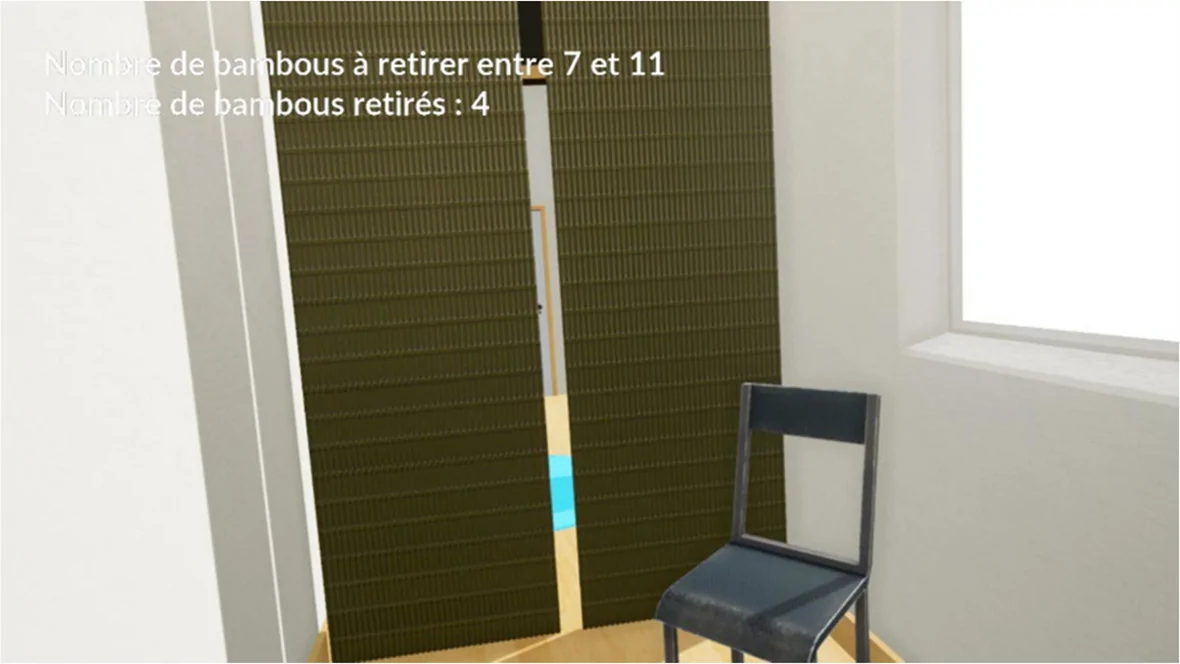
Zone 4

**Fig. 7:**
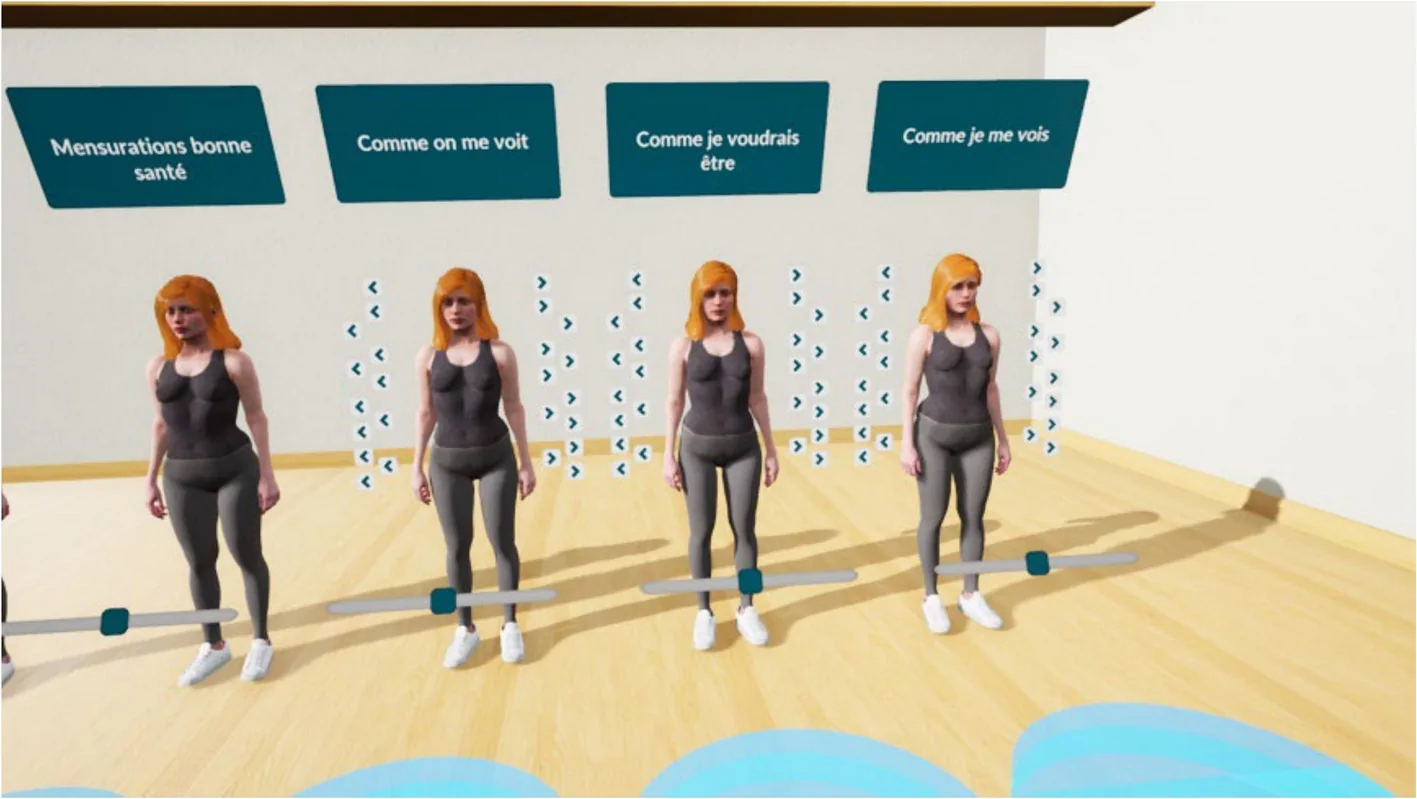
Zone 5

For this treatment, we will use a computer with our VR exposure program combined with a virtual reality headset, allowing 3D vision, and a pair of tracked controllers that provide intuitive hand presence in VR. An avatar representing the patient will be constructed from a number of measurements and morphological characteristics, such as skin color, hair length and color, height, and waist size (Fig. 2), so that the patient can identify herself. Five zones will be built in virtual reality, inspired by the environments of Perpiña, Botella, and Baños [27, 28]. Each zone will have the following objectives:

- 1st zone (Fig. 3) allows the patients to familiarize themselves with the controls of the VR device (move from one spot to another and interact with the environment).

- 2nd zone (Fig. 4): Patients were asked to create an avatar based on their perception of their own appearance. They then compared the constructed image with the actual body image, as determined by the therapist's measurements.

- 3rd zone (Fig. 5): to make the patients understand that “weight” alone does not have any meaning without other measures or variables, the patients have to assess the weight of 5 avatars with different morphotypes (height, body fat and lean mass…).

- 4th zone (Fig. 6): to have them evaluate their width by estimating the spacing required for it to pass through a doorway.

- 5th zone (Fig. 7): to develop an understanding of the differences between their idealized body, real body, and “healthy” body. In developing the healthy body used in this study, we relied on AFNOR standard NF ISO 8559–3 (“Size designation of clothes—Part 3: Methodology for creating body measurement tables and intervals”). This standard provides a statistical methodology to establish body measurement ranges based on anthropometric data from defined population groups (e.g., girls, boys, women, and men). In the context of our study of adolescent girls with AN, we used these guidelines to define a body shape that corresponds to a healthy, normative adolescent female corpulence. Therefore, the avatar’s anthropometric proportions were based on standardized body measurement intervals representative of a healthy population rather than on subjective aesthetic criteria.

The switch from one zone to another will be planned according to the sessions programmed in the protocol (with each session allowing work in one or two zones), and will evolve according to the points to be worked on, as identified by the therapist. All measurements and data collected from the patients will be listed in an electronic case report form (see Data Management).

Each VRET session will consist of three stages:

- Individual psychological interviews with cognitive behavioral conducted at the beginning of the session (20 min). The therapist uses the Socratic method, posing questions in the form of hypotheses to help patients synthesize information and draw conclusions.
- Immersion in a virtual environment (20 min). The therapist instructed patients on how to complete the task set in each zone. The patients were accompanied by a therapist who verbalized their emotions and automatic thoughts.
- They will then be guided through a critical analysis of these (20 min), focusing on cognitive counter-argumentation, which involves identifying errors in reasoning and replacing them with more accurate perceptions and interpretations. Participants will also be encouraged to identify the negative words used to describe the perceptions and situations experienced in these zones and link them to the associated emotions.

Each session lasted for 60 min. In the first “intensive” phase, there was 1 session every week from V2 to V10 then in the “maintenance” phase, there was 1 session every month from V10 to V18. V2 is a session in which the patient meets the therapist, receives an explanation of the treatment technique, and is introduced to the VRET device in zone 1. The eight sessions (V3–V10) comprising the intensive phase followed the plan described in the protocol. The transitions from one zone to another during the sessions are summarized in Table 2.

**Table 2:**
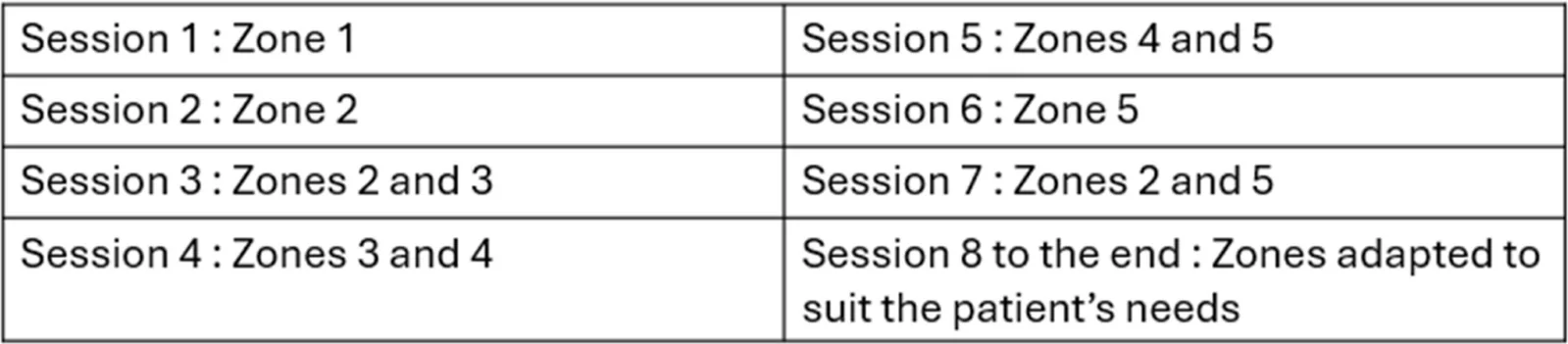
Zone transition

No other specific treatments for BD-SRC were administered during this study. The healthcare teams responsible for patients' routine care were informed of their inclusion in the research protocol. The participants were also informed of the need to adhere to this requirement.

### Body-first psychomotricity approach to treatment of perceptions/representation of “body contours”

This treatment focuses on body image perception and awareness as well as emotional regulation through the body. A device that engages the body and speech will be offered as part of the relaxation sessions for 60 min, which will be carried out identically across each session.

These sessions will be inspired by the works of Probst [28], Thornquist, and Bunkan [24]. A typical session is structured as follows.

- First, there was an initial speaking period aimed at establishing and maintaining a trusting relationship with the psychomotor therapist and reducing anxiety (20 min).
- The next step was body-centered relaxation and proprioceptive integration (20 min), which included the following:
  - Position and Postural Adjustment Exercises
  - Immobility after tonic mobilization exercises
  - Neuromuscular proprioceptive work: This phase increases awareness of body stability and the ability to relax.
  - The therapist provided verbal and physical instructions. A psychomotor therapist helps patients perceive bodily sensations and mental representations through verbal guidance and passive body mobilization
- Therapeutic support is provided in silence during the emergence of anxiety symptoms that may arise during the session.
  - The therapist progressively reactivates the body through active stretching movements.

- The session concludes with verbal expression, drawing, or modeling activities focused on the perception of “body boundaries,” which facilitate the progressive elaboration and integration of sensations and perceptions experienced during the session (20 min).

Each session will offer various realizations in accordance with the needs of each patient. Meetings were held regularly to ensure that psychomotor therapists used the same methods.

No other specific treatments for BD-SRC were administered during this study.

### Measures

#### Primary outcome

##### Body dissatisfaction and shape-related concerns

- The primary outcome will be the evolution of the overall Body Shape Questionnaire (BSQ) score between inclusion and 12-month assessment. The BSQ will be used to measure preoccupations with body shape and body dissatisfaction [26, 29 ]. The BSQ is a self-report unidimensional questionnaire with 34 items rated on a six-point Likert scale (from 1 “never” to 6 “always”). The total scores range between 34 and 204, with higher scores indicating significant concerns related to body image. The BSQ can be divided into four sub-scores: “avoidance and social embarrassment when exposing the body,” “physical dissatisfaction relating to the lower parts of the body,” “use of laxatives and vomiting to reduce physical dissatisfaction,” and “ill-adapted behaviors and cognitions in order to control weight.”
- The Contour Drawing Rating Scale (CDRS) is a self-report questionnaire comprising nine silhouettes shown on a continuum from silhouette 1, considered “extremely thin,” to silhouette 9, considered “extremely overweight” [30]. Each silhouette was shown according to the reference position (facing forward, legs slightly apart, and parallel, with the arms stretched out along the sides of the body, slightly apart). Patients must select the silhouette that they think currently resembles them, and the silhouette that represents their ideal body size. Body dissatisfaction is represented as the difference between the current and ideal sizes.

##### Anorexia nervosa

- The Eating Disorder Inventory (EDI-2) will be used to evaluate the severity and changes in ED symptoms [31]. The EDI-2 is a self-report questionnaire with 91 items rated on a 6-point Likert scale ranging from “always” to “never.” The EDI-2 consists of 11 dimensions: pursuit of thinness, bulimia, physical dissatisfaction, inadequacy, perfectionism, interpersonal distrust, interoceptive awareness, maturity fears, asceticism, impulse control, and social insecurity. Higher scores indicated greater severity of eating disorder symptoms and associated psychological difficulties. The EDI-2 is widely used in adolescent and clinical populations, and its psychometric properties, including high internal consistency, satisfactory test–retest reliability, and good construct validity, have been demonstrated to be good. In the present study, the EDI-2 will be used to evaluate changes in eating disorder symptoms throughout the intervention.
- Changes in body weight will enable us to assess the progression of AN. This will be analyzed primarily using the median BMI percentage (% mBMI = observed BMI × 100/expected median BMI for age), calculated using the World Health Organization (WHO) growth standards (https://www.who.int/tools/growth-reference-data-for-5to19-years/indicators/bmi-for-age). Raw BMI will be reported for descriptive purposes but will not be used as a primary criterion because of its dependence on age during adolescence.

##### Anxiety

The State-Trait Anxiety Inventory (STAI) is made up of two separate parts that independently evaluate traits and state anxiety [32]. Each part includes 20 items graded by four degrees according to their frequency or intensity. The scores for each component ranged from 20 to 80. Although no universal diagnostic cutoff exists, scores ≥ 39–40 are commonly considered indicative of clinically significant anxiety symptoms in non-clinical populations. The anxiety-trait scale is mostly used to evaluate anxious personality characteristics, whereas the anxiety-state scale measures changes brought about by various experimental situations. The STAI has demonstrated good psychometric properties, including high internal consistency, test-retest reliability, and construct validity.

##### Central coherence

The ROCF evaluates episodic memory using visual modalities and visuoconstructive abilities [33]. Furthermore, It is used to measure a variety of cognitive processes such as planning, organizational skills, problem-solving strategies, and central coherence, as well as perceptual and motor skills. It comprises 18 graphic elements that will be immediately reproduced. In this study, the ROCF will be used as an indirect measure of central coherence. Scoring will be performed according to Booth’s 2008 model. Higher central coherence index scores reflect a more global and integrated processing style, whereas lower scores indicate weaker central coherence and a tendency toward detail-focused processing.

##### Cognitive flexibility

The Trail Making Test (TMT) is an evaluation of executive functions, especially visuomotor skills and cognitive flexibility (Part B) [34]. The test was divided into two parts: Part A consisted of linking numbered circles in ascending order and Part B consisted of linking numbered circles alternately with circles containing a letter in ascending and alphabetic order. In this study, TMT Part B will be used to assess cognitive flexibility. Performance was evaluated using completion time and number of errors, with longer completion times and higher error rates indicating poorer cognitive flexibility and executive functioning.

##### Clinical data recorded

Sociodemographic data will include age, education level, family lifestyle (parents’ marital status, parents’ occupations, siblings, and birth order), and family medical history, including eating disorders. Data on AN history will include the age at which the disorder began, reported triggering factors, estimated weight before disorder onset, duration of evolution from weight before disorder onset to lowest weight, purging behavior, primary polydipsia, taking laxatives and/or anorectics, psychiatric comorbidities, and ED denial, if present. The Mini International Neuropsychiatric Interview for Children and Adolescents (MINI-KID) is a brief structured diagnostic interview. It is designed to assess DSM-IV and ICD-10 psychiatric disorders in children and adolescents aged 6–17. The MINI-KID provides a reliable and valid method for identifying a broad range of psychiatric conditions while requiring significantly less administration time than longer diagnostic interviews. Owing to its strong psychometric properties, including good reliability and concurrent validity, it is widely used in clinical and research settings [35]. The MINI-KID will be used to assess the DSM-based psychiatric diagnoses and associated comorbidities in adolescents with AN, including mood, anxiety, and behavioral disorders. It will also enable the evaluation of eating disorder diagnostic criteria, including the limited recognition of the severity of low body weight and related symptoms. This may reflect the denial of illness commonly observed in AN. However, this instrument was not specifically designed to assess insight or denial quantitatively. These assessments will complement the clinical data collected during the semi-structured interviews conducted at the start of the study.

Under French law governing the monitoring of clinical trials involving human subjects (https://www.legifrance.gouv.fr/codes/section_lc/LEGITEXT000006072665/LEGISCTA000006196156/), any adverse event occurring in a person taking part in such research will be recorded, “regardless of whether it is related to the research or the product being studied.” The adverse events expected in this study were as follows:

Risks of somatic and psychological complications of AN

- The use of VRET with the Phenomenon of “Simulator sickness” with three classes of symptoms: oculomotor (headaches, diplopia, blurred vision, visual fatigue), disorientation (dizziness, vertigo, postural instability, impaired psychomotor performance), nausea (drowsiness, increased salivation, sweating, loss of appetite, eructation, vomiting) and also a temporary increase in anxiety linked to confrontation with the bodily problems of AN through treatment.
- Psychomotricity treatment and the use of VRET with a temporary increase in anxiety linked to confrontation with bodily problems of AN through treatment.

### Data analysis

#### Data management

As soon as patients and their parents or guardians consent to participate, they will be included and given an ID number. An electronic case report form (eCRF) will be drawn up specifically for this study by a team of clinicians, methodologists, data managers, and statisticians. Electronic data will be saved on a secure server with restricted access.

#### Statistical methods

The data will be described using either means and standard deviations or medians and ranges for quantitative variables and using totals and percentages for qualitative variables. A comparison of the initial characteristics of the two groups will be conducted using Student’s *t*-tests, Mann–Whitney *U* test, Chi-square test, or Fisher’s exact test, according to the conditions for application.

Regarding the primary study outcome, the evolution of total BSQ scores between inclusion and 12 months post-randomization will be compared between the two treatment groups using Student’s *t*-test or the Mann–Whitney *U* test, according to the conditions of application.

For the secondary efficacy objectives, changes in the BSQ, EDI-2, and CDRS scores between baseline and the end of the intensive phase will be compared between the treatment groups using Student’s t-tests or Mann–Whitney U tests, as appropriate.

At Month 12, changes in BSQ subscale scores (“avoidance and social shame regarding body exposure,” “physical dissatisfaction with regard to the lower body,” and “inappropriate cognitions and behaviors to control weight”), as well as changes in total EDI-2 and CDRS scores, will be compared between treatment groups using *t*-tests or Mann–Whitney *U* tests depending on the data distribution.

For long-term outcomes, within-group changes in the BSQ, EDI-2, and CDRS scores will be assessed using paired Student’s *t*-tests or Wilcoxon signed-rank tests, depending on the data distribution.

Associations between changes in median BMI and long-term outcomes will be assessed using Pearson’s or Spearman’s correlation coefficients, as appropriate. When analyzed as a categorical variable, the median BMI percentage will be compared using Student’s *t*-test or the Mann–Whitney *U* test.

Associations among anxiety, mental flexibility, and long-term outcomes will also be assessed using Pearson’s or Spearman’s correlation coefficients, depending on the data distribution.

The level of significance for all analyses will be set at 0.05. All analyses will be conducted using SAS version 9.4 (SAS Inc., Cary, NC, USA).

#### Population for statistical analyses

After randomization, patients will remain in their original groups, and an efficiency analysis will be conducted with the modified intention to treat (MITT), considering that the patients have completed at least one treatment consultation. Additionally, a per-protocol analysis will be performed to evaluate effectiveness. The per-protocol population will include participants who attended at least seven of the ten sessions of the intensive phase, reflecting adequate adherence to the intervention protocol.

#### Missing data

The study will be carried out in such a way as to minimize the number of missing data. For each variable, the number and percentage of missing data will be described. In the presence of missing data, the patterns and proportions of missingness will first be explored descriptively. If the data are considered missing completely at random (MCAR) or missing at random (MAR), multiple imputations using chained equations (MICE) will be performed to minimize the bias and loss of statistical power correlated with complete-case analyses. The imputation model will include all the variables used in the main analyses, such as treatment allocation and relevant baseline covariates. Sensitivity analyses will be conducted to compare the complete-case and imputed analyses and to assess the robustness of the results. This approach aligns with the latest recommendations for addressing missing data in randomized controlled trials [36].

### Changes to the protocol

Several modifications have been implemented and are detailed in the protocol amendment dated January, 2023. This amendment was submitted to and approved by the ethics committee (see Ethical Statement). The modifications were as follows: the assessor was initially blinded to the randomization group, but blindness was lifted considering that the questionnaires were self-reported; therefore, this would not influence the outcome. Moreover, since the scales are self-report questionnaires, they will be administered at 2, 12, and 24 months by a professional from the child psychiatry department who will be able to accompany the administration of the scales, rather than by a child psychiatrist alone, as initially planned.

The diagnosis of any type of AN at < 3 months of age was extended to 6 months without specific management of BD-SRC. We considered that increasing the diagnostic delay would not reduce the quality of the study, the expected benefits, or the evaluation of the primary and secondary outcomes, and would represent additional support for the patients included, given the slow progression of AN and the difficulty of improving BD-SRC. Increasing the delay could also be a way to include more patients in this study.

There was possible variability in the rhythm of the sessions during the intensive and maintenance phases; however, this did not alter the effect of the treatment. During the intensive phase, patients were not allowed to miss more than one session. Important protocol modifications were communicated via mail or videoconferencing.

Although epilepsy and severe myopia were not explicitly defined as exclusion criteria in the original protocol, they will be systematically considered when enrolling participants. This approach, guided by clinical and methodological considerations, was intended to preserve the internal validity of the study.

### Study progress

This study will begin enrollment on May 20^th^, 2021, and the target enrollment of 40 patients will be completed by May 1^st^, 2027.

### Ethical approval

This protocol was approved by the Ethics Committee of the “Comité de Protection des Personnes” (CPP)). The protocol will be conducted according to the principles of the Declaration of Helsinki and will be in line with the Guidelines for Good Clinical Practice. The trial was registered at ClinicalTrials.gov: xxx. xxx. The file will be declared to the French Data Protection Authority (CNIL; baseline methodology: MR001).

## Discussion

This study aims to develop the use of VRET in adolescents diagnosed with AN, given its effectiveness in adults with the same pathology. Continuation of BD-SRC after treatment can be a risk factor for AN relapse, a condition with a severe evolutionary prognosis. As a result, a global, effective, and targeted treatment for BD-SRC in patients with AN could improve long-term evolutionary prognosis from a health risk perspective for morbidity and mortality.

This study has some limitations. First, patients with AN often lack insight into their illness or are in denial, making it difficult for them to accept this demanding protocol [37]. Second, the intensive phases are complicated for the patient and the assessor because of the level of investment required, with one session per week for 2 months. A lack of assessment of depressive symptoms that could be associated with BD-SRC was noted [38]. Finally, when interpreting the results, it will be important to consider the fact that epilepsy and severe myopia were not included in the initial exclusion criteria.

However, this study also shows strengths. This is the first study in an adolescent population to examine VRET as a means of improving BD-SRC (effectiveness at 12 months post-randomization) as an innovative intervention. The inclusion of a long-term follow-up extending to 24 months will provide valuable insights into the durability of treatment effects. Additionally, the detailed description of the VR protocol enhances reproducibility, whereas the study’s strong ethical and administrative framework supports transparency. All participants in the study will receive standardized treatment for AN in accordance with the rules for good practice recommended by the consensus conference of the National Authority for Health (HAS). Furthermore the follow-up strategy is well-defined, with the expectation of improvement/stability and long-term recovery from AN. The risk of patients being lost to follow-up may be affected by anxiety associated with each treatment method. The emergence of this symptom during the sessions considered an expected aspect of the therapeutic process and may facilitate progressive improvement in BD-SRC. However, the risk of loss to follow-up was considered when calculating the number of participants required for the study.

### Trial status

Protocol version 4, 12/01/2023, recruitment began in May 20^th^, 2021, and will be completed in May 1^st^, 2027.

## Supplementary Information

Supplementary Material 1

## Data Availability

The datasets generated and analyzed in this study are available from the corresponding author upon reasonable request.

## Abbreviations

AN: Anorexia Nervosa
BD-SRC: Body dissatisfaction and shape-related concerns
BMI: Body mass index
CDRS: Contour Drawing Rating Scale
EDI-2: Eating Disorder Inventory
ROCF: Rey–Osterrieth Complex Figure Test
STAI: Spielberger State-Trait Anxiety Inventory
TMT: Trail Making Test
ULN: Upper limit of normal
VRET: Virtual Reality Exposure Therapy

## References

[1] Van Eeden AE, van Hoeken D, Hoek HW. Incidence, prevalence and mortality of anorexia nervosa and bulimia nervosa. Curr Opin Psychiatry. 2021;34:515–24. 10.1097/YCO.0000000000000739. PMC8500372 34419970

[2] van Eeden AE, van Hoeken D, Hendriksen JMT, Hoek HW. Increase in incidence of anorexia nervosa among 10- to 14-year-old girls: a nationwide study in the Netherlands over four decades. Int J Eat Disord. 2023;56:2295–303. 10.1002/eat.24064. 37773004

[3] Datta N, Hossepian K, Xie I, Gurcan HY, Behr S, Pouliadi M, et al. Anorexia nervosa across the lifespan: a review of recent literature. Focus (Am Psychiatr Publ). 2024;22:269–77. 10.1176/appi.focus.20230037. PMC11231474 38988469

[4] Salari N, Heidarian P, Tarrahi MJ, Mansourian M, Canbary Z, Daneshkhah A, et al. Global prevalence of eating disorders in children: a comprehensive systematic review and meta-analysis. Ital J Pediatr. 2025;51:107. 10.1186/s13052-025-01958-0. PMC11974214 40197519

[5] Silén Y, Keski-Rahkonen A. Worldwide prevalence of DSM-5 eating disorders among young people. Curr Opin Psychiatry. 2022;35:362–71. 10.1097/YCO.0000000000000818. 36125216

[6] Sullivan PF. Mortality in anorexia nervosa. Am J Psychiatry. 1995;152:1073–4. 10.1176/ajp.152.7.1073. 7793446

[7] Haute Autorité de Santé. Anorexie mentale: Prise en charge [Online]; 2017. https://www.has-sante.fr//portail/jcms/c_985715/.

[8] Bruch H. Perceptual and conceptual disturbances in anorexia nervosa. Obstet Gynecol Surv. 1962;17:730–2. 10.1097/00006254-196210000-00037. 13873828

[9] Callaghan GM, Duenas JA, Nadeau SE, Darrow SM, Van der Merwe J, Misko J. An empirical model of body image disturbance using behavioral principles found in functional analytic psychotherapy and acceptance and commitment therapy. Int J Behav Consult Ther. 2012;7:16–24. 10.1037/h0100932. PMC3755378 23997716

[10] Diagnostic and statistical manual of mental disorders. 5th ed. Arlington, VA: American Psychiatric Association; 2013

[11] Diagnostic and statistical manual of mental disorders. 5th ed., text revision. Washington, DC: American Psychiatric Association; 2022

[12] Stonawski V, Sasse L, Derks L, Moll GH, Kratz O, Legenbauer T, et al. Transdiagnostic body dissatisfaction: comparing adolescents with anorexia nervosa and depression during body exposure. Child Adolesc Psychiatry Ment Health. 2025;19:76. 10.1186/s13034-025-00939-9. PMC12257810 40660352

[13] Griffiths S, Murray SB, Touyz S. Drive for muscularity and muscularity-oriented disordered eating in men: the role of set shifting difficulties and weak central coherence. Body Image. 2013;10:636–9. 10.1016/j.bodyim.2013.04.002. 23680082

[14] Chong KK, Martinelli C. Weak central coherence, cognitive rigidity and disordered eating in a community sample. Eat Behav. 2024;53:101866. 10.1016/j.eatbeh.2024.101866. 38452626

[15] Ferrer-García M, Gutiérrez-Maldonado J. The use of virtual reality in the study, assessment, and treatment of body image in eating disorders and nonclinical samples: a review of the literature. Body Image. 2012;9:1–11. 10.1016/j.bodyim.2011.10.001. 22119329

[16] Serino S, Polli N, Riva G. From avatars to body swapping: the use of virtual reality for assessing and treating body-size distortion in individuals with anorexia. J Clin Psychol. 2019;75:313–22. 10.1002/jclp.22724. PMC6590458 30552669

[17] Porras-Garcia B, Serrano-Troncoso E, Carulla-Roig M, Soto-Usera P, Ferrer-Garcia M, Figueras-Puigderrajols N, et al. Virtual reality body exposure therapy for anorexia nervosa. A case report with follow-up results. Front Psychol. 2020;11:956. 10.3389/fpsyg.2020.00956. PMC7242758 32499742

[18] Marco JH, Perpiñá C, Botella C. The treatment of the body image disturbances in eating disorders and clinically significant change. Ann Psychol. 2013;30:422–30.

[19] Porras-Garcia B, Ferrer-Garcia M, Serrano-Troncoso E, Carulla-Roig M, Soto-Usera P, Miquel-Nabau H, et al. AN-VR-BE. A randomized controlled trial for reducing fear of gaining weight and other eating disorder symptoms in anorexia nervosa through virtual reality-based body exposure. J Clin Med. 2021;10:682. 10.3390/jcm10040682. PMC7916489 33578767

[20] Ascione M, Carulla-Roig M, Meschberger-Annweiler F-A, Serrano-Troncoso E, Blasco-Martínez A, Guerrero-Álvarez F, et al. Virtual reality body exposure and attentional bias modification in the treatment of adolescents with anorexia nervosa. Clin Psychol Psychother. 2026;33:e70273. 10.1002/cpp.70273. PMC13078948 41980806

[21] Mares SHW, Voskamp M, Wezenberg E, van Elburg AA. A feasibility study of an add-on psychomotor body-image protocol during CBT-E in female patients with an eating disorder. J Behav Ther Exp Psychiatry. 2025;88:102034. 10.1016/j.jbtep.2025.102034. 40179772

[22] Probst M. Psychomotor therapy for patients with severe mental health disorders. In: Occupational therapy - occupation focused holistic practice in rehabilitation. InTech; 2017.

[23] Young C. The history and development of body-psychotherapy: the American legacy of Wilhelm Reich1. Body Mov Dance Psychother. 2008;3:5–18. 10.1080/17432970701717783.

[24] Thornquist E, Bunkan BH. What is psychomotor physiotherapy? Oslo: Universitetsforl; 1991.

[25] Lucas G, Sin J, Cini E, Röhricht F. Body-oriented therapies for the treatment of eating disorders: a systematic review. Complement Ther Clin Pract. 2025;60:101997. 10.1016/j.ctcp.2025.101997. 40393177

[26] Lavoisy G, Guelfi J-D, Vera L, Dardennes R, Rouillon F. Évaluation des préoccupations corporelles dans les troubles des conduites alimentaires par le Body Shape Questionnaire. Encephale. 2008;34:570–6. 10.1016/j.encep.2007.11.005. 19081453

[27] Perpiñá C, Botella C, Baños RM. Virtual reality in eating disorders. Eur Eat Disord Rev. 2003;11:261–78. 10.1002/erv.520.

[28] Probst M. Physiotherapy in eating disorders: changes in body avoidance and body checking behavior over six months. Physiotherapy (Lond). 2015;101:e1238. 10.1016/j.physio.2015.03.1137.

[29] Rousseau A, Knotter R-M, Barbe R-M, Raich R-M, Chabrol H. Étude de validation de la version française du Body Shape Questionnaire. Encephale. 2005;31:162–73. 10.1016/S0013-7006(05)82383-8. 15959443

[30] Thompson MA, Gray JJ. Development and validation of a new body-image assessment scale. J Pers Assess. 1995;64:258–69. 10.1207/s15327752jpa6402_6. 7722852

[31] Thiel A, Paul T. Test–retest reliability of the Eating Disorder Inventory 2. J Psychosom Res. 2006;61:567–9. 10.1016/j.jpsychores.2006.02.015. 17011367

[32] Spielberger CD, Gorsuch RL, Lushene RE, Vagg PR, Jacobs G. Manual for the State–Trait Anxiety Inventory (form Y1—Y2). 1983;IV.

[33] Lang K, Roberts M, Harrison A, Lopez C, Goddard E, Khondoker M, et al. Central coherence in eating disorders: a synthesis of studies using the Rey Osterrieth Complex Figure Test. PLoS ONE. 2016;11:e0165467. 10.1371/journal.pone.0165467. PMC5091879 27806073

[34] Lezak MD, editor. Neuropsychological assessment. 5th ed. New York: Oxford University Press; 2012.

[35] Sheehan DV, Sheehan KH, Shytle RD, Janavs J, Bannon Y, Rogers JE, et al. Reliability and validity of the Mini International Neuropsychiatric Interview for Children and Adolescents (MINI-KID). J Clin Psychiatry. 2010;71:313–26. 10.4088/JCP.09m05305whi. 20331933

[36] Sterne JAC, White IR, Carlin JB, Spratt M, Royston P, Kenward MG, et al. Multiple imputation for missing data in epidemiological and clinical research: potential and pitfalls. Br Med J (Clin Res Ed). 2009;338:b2393. 10.1136/bmj.b2393. PMC2714692 19564179

[37] Gawron LP, Garrido-Ribas I, Carmona-Farrés C, Carril-Batuecas S, Carceller-Sindreu M, Avila-Parcet A, et al. Understanding the structure of insight in eating disorders: validation of the SAI-ED in a Spanish clinical sample. J Eat Disord. 2025;13:184. 10.1186/s40337-025-01360-5. PMC12376702 40855569

[38] Chen G, Guo G, Gong J, Xiao S. The association between body dissatisfaction and depression: an examination of the moderating effects of gender, age, and weight status in a sample of Chinese adolescents. J Psychol Counsell Sch. 2015;25:245–60. 10.1017/jgc.2015.6.

